# The Epidemiology of Non-Melanocytic Benign and Malignant Skin Tumors in Pediatric Patients Attending to the Dermatology Department

**DOI:** 10.14740/jocmr2190w

**Published:** 2015-08-23

**Authors:** Gulsen Tukenmez Demirci, Guldehan Atis, Ilknur Kivanc Altunay, Damlanur Sakiz

**Affiliations:** a Dermatology Department, Baskent University Istanbul Hospital, Istanbul, Turkey; b Dermatology Department, Siyami Ersek Cardiovasculer Surgery Hospital, Istanbul, Turkey; c Dermatology Department, Sisli Etfal Training and Research Hospital, Istanbul, Turkey; d Pathology Department, Bakirkoy Sadi Konuk Training and Research Hospital, Istanbul, Turkey

**Keywords:** Pyogenic granuloma, Nevus sebaceous, Pilomatricoma

## Abstract

**Background:**

Non-melanocytic skin tumors are rarely seen in pediatric patients; although they are mostly benign, they remain to be elucidated by histopathological examination. The objective of the study was to describe the epidemiology of non-melanocytic skin tumors in children attending to our dermatology department.

**Method:**

The histopathologic studies of all skin punch and excisional biopsies of children up to 16 years old referred to our dermatology department between January 2007 and January 2012 were reviewed retrospectively. Melanocytic tumors and cystic and infectious lesions were excluded. Age, sex, location, and histopathologic diagnosis were recorded. The skin tumors were categorized.

**Results:**

A total of 4,126 skin tumors were analyzed histopathologically, and 203 of the lesions were from children up to 16 years of age. Ninety-seven of the lesions from 91 patients were non-melanocytic skin tumors. Forty-seven (51.64%) were male, 44 (48.36%) were female, and mean age was 10.55 ± 4.31 years. Malignant tumor was 1.03% (one tumor) and benign tumors were 98.97% (96 tumors) of all. The most frequent non-melanocytic skin tumor was pilomatricoma with 22 lesions (22.68%), followed by pyogenic granuloma with 18 lesions (18.54%), and nevus sebaceous with 10 (10.3%) lesions. Cutaneous leukemic infiltrate was found to be the only malignant skin tumor in the study group. The most frequently affected age group was children aged > 13 to ≤ 16 years, which included 38 patients (41.7%). The majority of lesions were on head and scalp (32 tumors, 32.96%), followed by trunk (28 tumors, 28.84%) and upper limbs (22 tumors, 22.75%).

**Conclusion:**

The ratio of malignant to benign skin tumors in pediatric patients is found to be small. Pilomatricoma, pyogenic granuloma and nevus sebaceous are found to be the most frequent non-melanocytic skin tumors of children. The ratio of malignant tumors is very rare.

## Introduction

The skin of children has different histologic, physiologic, and immunologic characteristics than the skin of adults. Pediatric dermatology is therefore an emerging and growing specialty within the fields of pediatrics and general dermatology [1]. Malignant tumors, such as basal cell carcinoma, squamous cell carcinoma, and melanoma, which are the most common malignant skin tumors in adulthood, are very rare in childhood [2]. Only 1-2% of the skin tumors excised from children are malignant [3]. Although most of the skin tumors seen in children are benign, histopathological confirmation is needed for an exact diagnosis if the lesions are non-melanocytic and there are no other diagnostic approaches. In this study, we aimed to perform an epidemiologic survey of non-melanocytic skin tumors that were biopsied or excised from patients aged 16 years or younger at the Dermatology Department of Sisli Etfal Training and Research Hospital, in Istanbul, over a period of 5 years.

## Materials and Methods

The study was performed in the Dermatology and Pathology Departments at Sisli Etfal Training and Research Hospital in Istanbul. From the database of the Pathology Department, we extracted all histopathology reports of patients aged 16 years old and younger with skin tumors who had been referred to dermatology department from January 2007 to January 2012 (a 5-year period). Melanocytic tumors and cystic and infectious lesions were excluded. The age, sex, location of the tumor, and histopathologic diagnoses of the patients were recorded. The non-melanocytic skin tumors were categorized as skin adnexal tumors; vascular tumors; tumors and tumor-like proliferations of fibrous and related tissues; non-lymphoid cutaneous infiltrates; tumors of muscle, cartilage, and bone; tumors of the epidermis; neural and neuroendocrine tumors; tumors of fat; and cutaneous metastases. The most common skin tumors were also grouped according to the age and sex of the patient and the location of the tumor.

## Results

We found that 203 (4.92%) of the 4,126 skin tumors were from children up to 16 years of age, and 97 (47.78%) skin tumors, from 91 patients, were non-melanocytic, while 106 (52.22%) of the lesions were diagnosed as melanocytic, cystic, or infectious. Among the patients, 47 (51.64%) were male, while 44 (48.36%) were female. The mean age was 10.37 ± 4.37 years.

**Figure 1:**
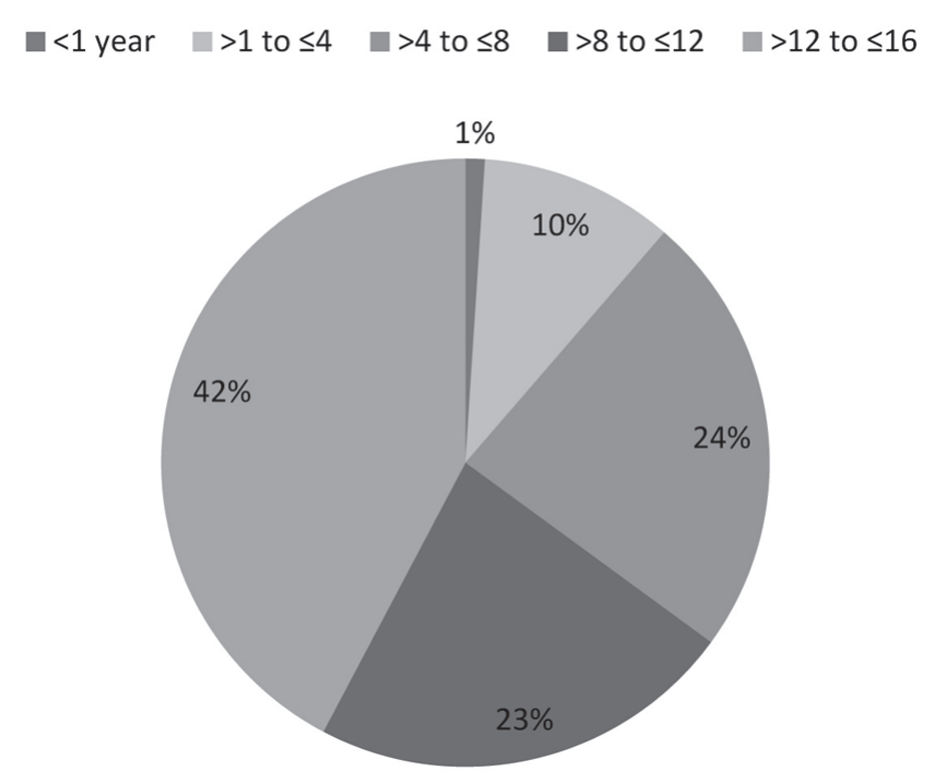
Distribution of non-melanocytic skin tumors according to age group.

Thirty-eight skin tumors (41.7%) were found in patients aged > 13 to ≤ 16 years, 21 (23.1%) skin tumors were found in patients aged > 8 to ≤ 12 years, 22 (24.2%) were found in patients aged > 4 to ≤ 8 years, nine (9.9%) were found in patients aged > 1 to ≤ 4 years, and one (1.1%) was found in a patient under 1 year of age (Fig. 1).

**Figure 2:**
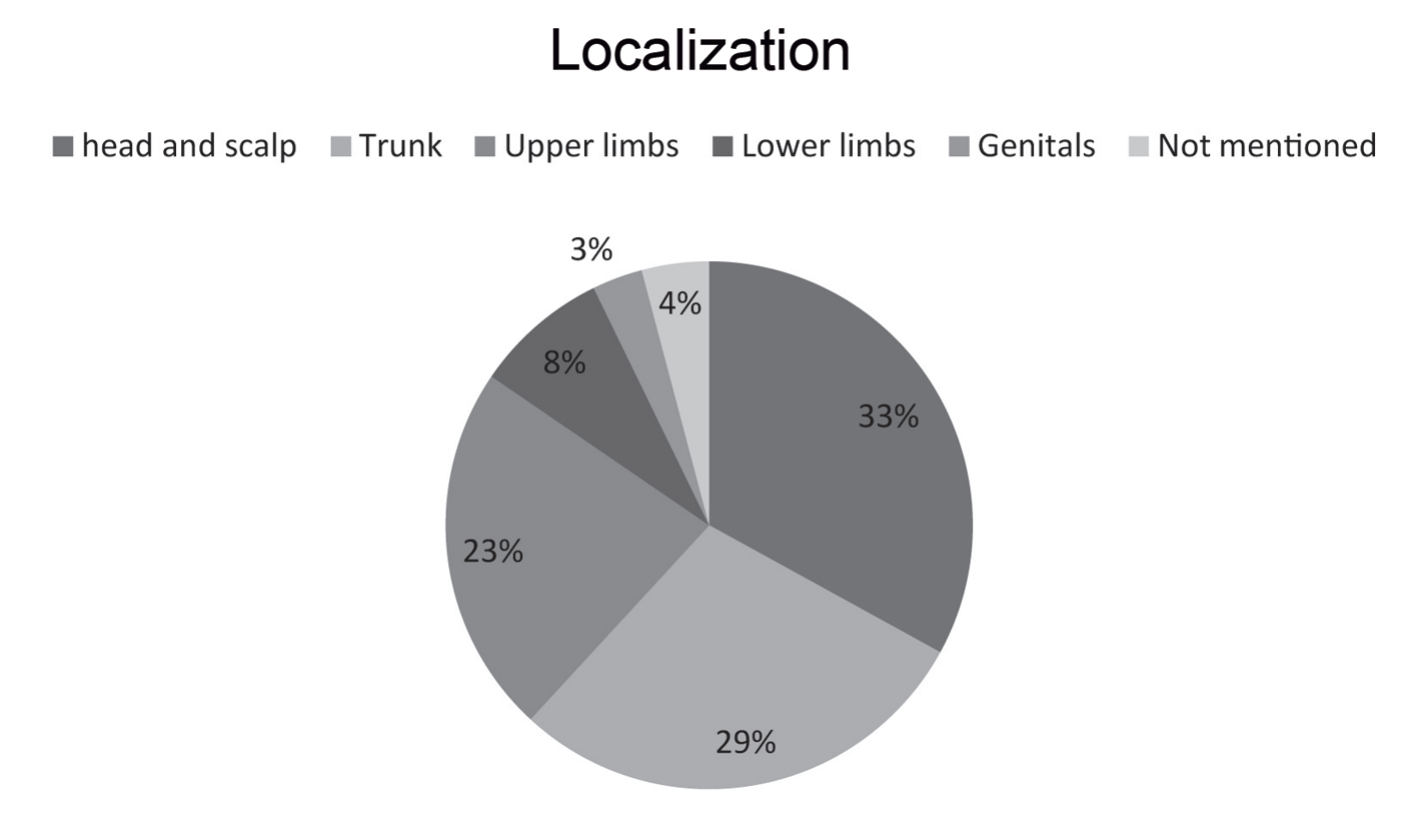
The location of non-melanocytic skin tumors.

The most common biopsy site was the head and scalp (n = 32, 32.96%), followed by the trunk (n = 28, 28.84%), the upper limbs (n = 22, 22.75%), the lower limbs (n = 8, 8.24%), the genitals (n = 3, 3.9%), or the site that was not mentioned (n = 4, 4.12%) (Fig. 2).

The five most common histopathologic diagnoses were pilomatricoma (22 tumors, 22.68%), pyogenic granuloma (18 tumors, 18.54%), nevus sebaceous (10 tumors, 10.3%), fibroepithelial polyp (six tumors, 6.18%), and juvenile xanthogranuloma (five tumors, 5.15%). The most commonly diagnosed histopathological group was adnexal tumors. The most common diagnoses in this group were pilomatricoma and nevus sebaceous. The second most commonly diagnosed group was vascular tumors. Pyogenic granuloma and capillary hemangioma were the most frequent diagnosis in this group. A cutaneous leukemic infiltrate was the only malignant skin tumor in the study group (1.03%). The most common diagnoses by age group are shown in Table 1.

**Table 1:** Histopathological Group and Histologic Diagnosis of Non-Melanocytic Skin Tumors According to Age Group

Age	Histopathological group	Histologic diagnosis	Number of patients
< 1 year	Non-lymphoid cutaneous infiltrates	Juvenile xanthogranuloma	1
> 1 to ≤ 4 years	Vascular tumors	Capillary hemangioma	1
		Pyogenic granuloma	1
		Lymphangioma	1
	Non-lymphoid cutaneous infiltrates	Juvenile xanthogranuloma	2
	Skin adnexal tumor	Pilomatricoma	1
	Tumors of fat	Lipoma	1
	Tumors and tumor-like proliferations of fibrous and related tissues	Fibroepithelial polyp	1
	Tumors of muscle, cartilage, and bone	Congenital midline hamartoma	1
> 4 to ≤ 8 years	Vascular tumors	Lymphangioma	2
		Capillary hemangioma	2
		Pyogenic granuloma	1
		Venous hemangioma	1
		Cavernous hemangioma	1
		Angiokeratoma	1
	Skin adnexal tumors	Pilomatricoma	2
		Syringocystadenoma papilliferum	1
	Tumors of epidermis	Epidermal nevus	1
		Inflammatory linear verrucous epidermal nevus	1
		Seborrheic keratosis	1
	Tumors and tumor-like proliferations of fibrous and related tissues	Fibrous hamartoma of infancy	1
		Fibroepithelial polyp	1
	Non-lymphoid cutaneous infiltrates	Juvenile xanthogranuloma	1
		Mastocytosis	1
	Tumors of muscle, cartilage, and bone	Chondroma	2
	Neural and neuroendocrine tumors	Neuroma	2
	Cutaneous metastases	Acute myeloid leukemia	1
> 8 to ≤ 12 years	Skin adnexal tumors	Pilomatricoma	11
		Sebaceous nevus	1
	Vascular tumors	Pyogenic granuloma	6
		Capillary hemangioma	1
	Tumors and tumor-like proliferations of fibrous and related tissues	Fibroepithelial polyp	2
		Fibrohistiocytic tumor	1
> 12 to ≤ 16 years	Skin adnexal tumor	Sebaceous nevus	9
		Pilomatricoma	8
		Clear cell hidradenoma	1
	Vascular tumors	Pyogenic granuloma	10
		Lymphangioma circumscriptum	2
		Cavernous hemangioma	1
		Arteriovenous hemangioma	1
	Tumors and tumor-like proliferations of fibrous and related tissues	Dermatofibroma	3
		Fibroepithelial polyp	2
	Tumors of epidermis	Epidermal nevus	1
	Tumors of fat	Lipofibroma	1
	Neural and neuroendocrine tumors	Neurofibroma	1
	Tumors of muscle, cartilage, and bone	Leiomyoma	1

There were five boys and 13 girls with pilomatricoma, and their mean age was 10.88 ± 3.42 years. The pilomatricomas were located on the upper limbs (13), the trunk (three), the head and scalp (five), or the location that was not mentioned (one). Among these patients, 16 (88.89%) had single lesions, while two patients (11.11%) had multiple lesions (Table 2).

**Table 2:** The Mean Age and Gender of the Patients and the Location of the Pilomatricoma, Pyogenic Granuloma, and Nevus Sebaceous Tumors

	Pilomatricoma	Pyogenic granuloma	Nevus sebaceous
Mean age	10.88 ± 3.42	11.66 ± 3.83	14.11 ± 2.10
Gender			
Female	13 (72.22%)	6 (33.33%)	3 (30%)
Male	5 (27.78%)	12 (66.66%)	7 (70%)
Location			
Head and scalp	5 (22.72%)	8 (44.44%)	10 (100%)
Trunk	3 (13.62%)	7 (38.88%)	-
Upper limbs	13 (59.12%)	2 (11.11%)	-
Lower limbs	-	-	-
Genitals	-	1 (5.5%)	-
Not mentioned	1 (4.54%)	-	-

The mean age of the children (12 males and six females) with pyogenic granuloma was 11.66 ± 3.83 years. The pyogenic granulomas were located on the head and scalp (eight), the trunk (seven), the upper limbs (two), and the genitals (one) (Table 2).

There were seven males and three females with nevus sebaceous. Their mean age was 14.11 ± 2.10 years. All the nevus sebaceous lesions were located on the head and scalp (Table 2).

## Discussion

We found that 203 of the 4,126 histopathologically diagnosed skin tumors occurred in pediatric patients up to 16 years old, and these cases were used in our study. Thus, the percentage of the total skin tumors from the pediatric patients (4.92%) was found to be small in comparison with the adult population presenting to our dermatology department. We excluded melanocytic, cystic, and infectious lesions in order to define the authentic frequency and epidemiology of the more uncommon non-melanocytic skin tumors in children. In the study by Lopez et al [1], melanocytic tumors were the most frequent diagnosis, followed by pilomatricoma. We also found that pilomatricoma was the most common skin tumor (after excluding melanocytic lesions), which matched the study by Lopez et al.

Pilomatricoma is the most common benign skin tumor in pediatric patients, and it occurs mainly in the head and neck region [4, 5]. Lucas et al reported on non-melanocytic benign skin tumors in their study and found that pilomatricoma was the most frequent benign skin tumor [6]. Guinot-Moya et al found that pilomatricoma tends to present in children and that it has a slight male predilection. Only 2.42% of cases presented multiple lesions, nearly all the cases presented as single lesions, and the most common localizations were the head and orofacial zones [7]. In a retrospective study, out of a total of seven patients, five (71%) were female and two (29%) were male [8]. In our study, we found that 16 patients (88.89%) had single lesions and two patients (11.11%) had multiple lesions. Among these patients, 13 (72.22%) were female, whereas five (27.78%) were male. The most common localization was the upper limbs, followed by the head and scalp and the trunk.

Pyogenic granuloma is another common skin tumor in children. These vascular-papular, rapidly growing erythematous lesions develop most commonly on the head, neck, extremities, and fingers [9]. We found that pyogenic granuloma was the second most frequent non-melanocytic skin tumor in our study. In a retrospective study conducted by Pagliai et al, 128 children had a clinical diagnosis of pyogenic granuloma, and the locations of the lesions were reported for 108 patients, with the most common locations for pyogenic granulomas being the head and neck (76.9%), followed by the chest and back (11.9%), the upper extremities, and the lower extremities (2.8%) [10]. In our study, the most common location for pyogenic granulomas was the head and scalp (eight patients, 44.44%), followed by the trunk (seven patients, 38.88%), the upper limbs (two patients, 11.11%), and the genitals (one patient, 5.5%). Our results are similar to their study.

Nevus sebaceous was the third most common non-melanocytic skin tumor in our study. Ten patients had nevus sebaceous tumors, and all of these were located on the head and scalp. Nevus sebaceous is a kind of classical nevus or congenital hamartomatous disorder. The prevalence of nevus sebaceous is 0.3% [11]. More than 90% of sebaceous nevi involve the head, mainly on the scalp, and such lesions present at birth. In 10-30% of cases, secondary skin neoplasms develop on the nevus, usually in adulthood [12, 13]. No other skin neoplasms were found on the nevus sebaceous in our patients, as our study population comprised young patients under the age of 16.

We found only one patient (1.03%) with a malignant non-melanocytic skin tumor in our study. In their study, Hamm et al reported that only 1-2% of all skin tumors excised from infants and children were malignant [12]. The single patient with a malignant skin tumor was a 5-year-old boy with leukemia cutis who was diagnosed with acute non-lymphocytic leukemia (ANLL). In a retrospective study, Tsukimoto showed that cutaneous leukemia developed in 15.9% of all patients with ANLL, and Tsukimoto suggested that cutaneous leukemia tends to occur early in infants with ANLL [14].

### Conclusion

In conclusion, the ratio of malignant to benign skin tumors in pediatric patients is found to be low. Children aged > 13 to ≤ 16 years had the highest ratio. The majority of lesions were on the head and scalp. Pilomatricoma, pyogenic granuloma, and nevus sebaceous were found to be the most frequent non-melanocytic benign skin tumors. Although malignant tumors are very rare, histopathological examination is still necessary for confirmation.
